# Unraveling the association of bacteria and urinary stones in patients with urolithiasis: an update review article

**DOI:** 10.3389/fmed.2024.1401808

**Published:** 2024-08-30

**Authors:** Abdolah Razi, Azita Ghiaei, Fahimeh Kamali Dolatabadi, Ramin Haghighi

**Affiliations:** ^1^Department of Urology, Faculty of Medicine, North Khorasan University of Medical Sciences, Bojnurd, Iran; ^2^Department of Microbiology, Isfahan (Khorasgan) Branch, Islamic Azad University, Isfahan, Iran; ^3^Department of Cell and Molecular Biology, School of Biology, College of Science, University of Tehran, Tehran, Iran

**Keywords:** urolithiasis, urinary stone, urinary tract infection, bacterial infection, microbium urolithiasis, microbium

## Abstract

Urinary stone disease (USD) is a prevalent urological condition, ranking as one of the most common urinary tract disorders globally. Various risk factors influence the formation of kidney stones, and recent research indicates a rising prevalence of urolithiasis worldwide, particularly in developing countries. While the morbidity associated with urinary stones has decreased in recent years, long-term complications such as stone recurrence, kidney failure, and uremia continue to burden patients. Understanding the etiologies of urolithiasis, including the role of bacteria, is crucial as they can contribute to stone recurrence. The incidence of urinary tract infection (UTI) stones can be attributed to specific infectious risk factors, socio-demographic factors, and comorbid metabolic disorders. This review article explores the emerging evidence suggesting the involvement of bacteria in USD. It discusses the potential role of microorganisms in non-infection stones and highlights the association between UTIs and urolithiasis. Furthermore, it surveys the relationship between kidney stones and recurrent UTIs and the formation of bacterial biofilms in UTIs. Considering various risk factors, including biochemical stone analysis and the presence of bacteria, is essential for treating patients with infectious stones optimally. This review aims to provide an updated understanding of the association between bacteria and urinary stones in patients with urolithiasis, shedding light on the pathophysiology of urinary stone formation, urinary stone characteristics, and the urinary microbiome in urinary stones.

## Introduction

Urinary stone disease (USD), which encompasses various terms such as nephrolithiasis, urolithiasis, nephrocalcinosis, and kidney stones, is a prevalent urological condition. Globally, it ranks as the third most prevalent disorder of the urinary tract, trailing urinary tract infections (UTIs) and benign prostate hypertrophy (1–3). USD is multifactorial, and various risk factors can influence the formation of kidney stones (4). Emerging research indicates a rising prevalence of urolithiasis all around the world with an upward trend in developing countries (5). The prevalence of urolitiasis is approximately 5%, 9%, 10%, 13% in Asia, Europe, United States, South America, respectively; and remarkably high at 42.9% in Sub-Saharan Africa (6). Long-term complications such as the risk of stone recurrence, kidney failure, and uremia continue to impose a significant burden on patients in the future (7).

**FIGURE 1:**
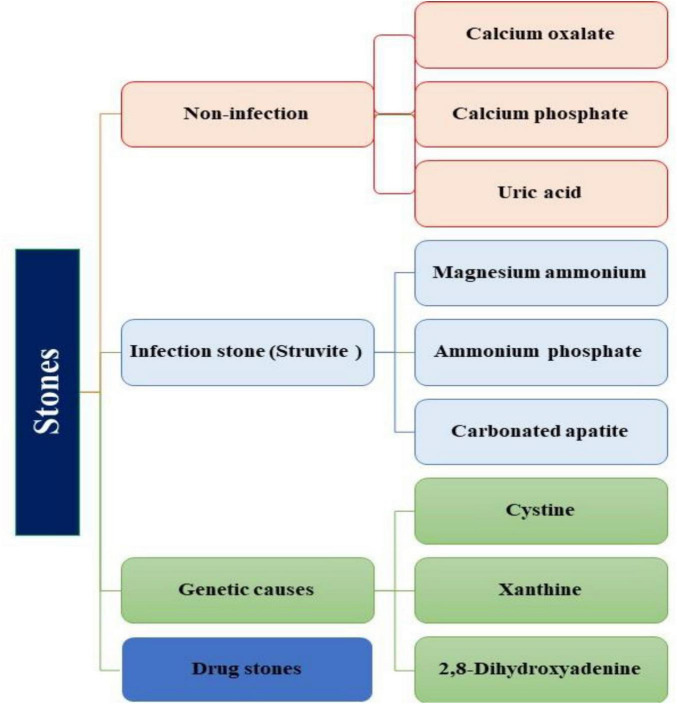
Stones classified by etiology.

Urinary stones can be classified into four types based on their formation and composition: I) Non-infection stones (Calcium oxalate (CaOx), Calcium phosphate (CaP), Uric acid); II) Infection stones (Magnesium Ammonium phosphate, Carbonate apatite, Ammonium urate); III) Genetic defects (Cystine, Xanthine, 2, 8-dihydroxyadenine); IV) Adverse drug effects (drug stones) (5, 8) (Figure 1). Since, urolithiasis significantly impacts the quality of life for affected individuals, it becomes crucial to comprehend the various etiologies, including the role of bacteria associated with urolithiasis, as they can potentially contribute to stone recurrence (9). The incidence of UTI stones in adult males exhibits variability, with relative proportions ranging from 3.2% to 10.1%. Notably, after the age of 50, the occurrence of UTI stones progressively increases (6). The prevalence of urolithiasis increases with age, with a breakdown of 5.1% in individuals aged 20–39 years, 11.5% in men aged 40–59 years, followed by 18.8% in men aged 60–79 years, and 19.7% in male individuals older than 80 years (4, 9). Infection-related stones, such as magnesium-ammonium-phosphate (struvite) calculi, are more prevalent in women than men of all ages. These disparities can be attributed to specific infectious risk factors present in certain populations, including factors like nutrition, access to modern medicine, the emergence of antibiotic-resistant bacteria, limited healthcare access, age, gender, body mass index (BMI), underlying diseases, dietary, genetic, and lifestyle (5, 10, 11). Socio-demographic factors and comorbid metabolic disorders demonstrated an association with USD. It has been suggested that type 2 DM (T2DM) may play a role in the formation of uric acid stones due to insulin resistance, which reduces urinary PH. Overweight and hypertension have been associated with CaOx stones (12). Overweight patients, in particular, are at an elevated risk of developing uric acid stones. Furthermore, patients with diabetes and hypertension had a higher frequency of uric acid stone (9, 13). Therefore, it is essential to consider various risk factors in conjunction with biochemical stone analysis when managing patients with infectious stones for optimal treatment. This review aims to provide an updated understanding of the association between bacteria and urinary stones in patients with urolithiasis, shedding light on the pathophysiology of urinary stone formation, urinary stone characteristics, and the urinary microbiome in urinary stones.

## Pathophysiology of urinary stone formation

In a healthy individual, urine usually contains substances that inhibit nucleation, crystal growth, aggregation, and adhesion of crystals to cells. These inhibitory chemicals adhere to the surface of crystals and play a crucial role in reducing the risk of developing urinary stones (14). These inhibitors exist in various forms, including inorganic anions such as pyrophosphates, organic anions like citrate, multivalent lic cations including magnesium, and macromolecules such as urinary prothrombin fragment-1, heparin, chrondrotion, osteopontin, glycosaminoglycans, glycoproteins and Tamm-Horsfall protein (5, 14). However, these inhibitors do not appear to exhibit uniform efficacy across all individuals; consequently, certain individuals may still develop stones. Furthermore, the abnormal function and concentration of these elements may contribute to the formation of stones (15).

Generally, tiny crystals are excreted through the urinary tract without causing noticeable problems (16). In contrast, certain substances known as promoters actively facilitate the formation of stones through various mechanisms. These promoters include cell-membrane lipids like cholesterol, glycolipids, and phospholipids. The calcitriol, stimulated by parathyroid hormone, also acts as a promoter. Other promoters include oxalate, cystine, calcium, sodium, and low urine volume. Studies have shown that individuals with recurrent stone formation have higher urinary oxalate excretion and lower citrate excretion, indicating an imbalance between stone inhibitors and promoters as a common cause of stone formation (16, 17).

Among the various types of urinary stones, non-infection stones are the most common in the general population. These stones are primarily formed due to excess stone-forming calcium salts in the urine, leading to a condition called supersaturation. Several factors contribute to the formation of non-infection stones, including hypercalciuria, which involves excessive calcium excretion in the urine. Hypocitraturia is another factor characterized by low levels of citrate in the urine, and citrate plays a role in inhibiting the growth and aggregation of calcium crystals. Hyperoxaluria, which refers to the excessive excretion of oxalate in the urine, is also involved, and hyperuricosuria is another contributing factor (18). Furthermore, uric acid stones are commonly found in their pure forms, either as anhydrous or dihydrate crystals. However, a small proportion, less than 1%, of uric acid stones may be mixed with other components such as ammonium acid urate or monosodium urate combined with CaOx (19, 20).

The appearance of struvite crystals can vary significantly in terms of their shape, and this is influenced by several factors related to their growth. These factors include PH, ion concentrations, crystal formation duration, substance proportions, and temperature (19, 21). Researchers have identified various morphological types of struvite crystals, which include prismatic, pyramidal, rectangular platelet, elongated platelet, dendritic forms, X-shaped, star-shaped, coffin-lid, and needle-shaped structures. These different crystal shapes result from variations in the sequence of noncovalent and ionic interactions, rearrangements, and ions clustering during the crystal formation process (22). The crystallization process is influenced by factors such as PH, temperature, residence time, and the dynamics of ionic interactions, all contributing to the wide range of observed crystal morphologies. Crystal morphology can be altered due to impurities, PH changes, and the presence of ions. Struvite crystals typically form within a PH range of 7 to 11, with the majority forming between 8 and 9 (23).

However, studies have shown that struvite can form under specific conditions such as high concentrations of ammonium and phosphate, low PH levels, and low magnesium concentrations. Also, the characteristics of infection stones vary depending on the presence or absence of bacteria (5, 24). In contrast to crystals formed in the absence of bacteria, bacteria result in the formation of struvite crystals and affect the PH levels. Elevated urine PH levels promote the accumulation of NH4+, CO32−, PO43− ions, and magnesium ions, all of which contribute to the crystallization of struvite. These struvite stones have the potential to grow rapidly and, if left untreated, can fill the intrarenal collecting system, posing a significant risk of kidney damage (25). Furthermore, research has indicated that PH changes induced by bacteria that produce urease can form struvite crystals in a dendritic form, often taking the shape of an X, particularly when PH levels shift rapidly (26).

Furthermore, bacteria increase the porosity of struvite stones, making them softer and more fragile. Due to their porous nature, struvite stones are relatively soft and fragile, which allows them to be easily fragmented during treatment. However, this can also release infectious bacteria into the urinary tract, increasing reinfection and recurrence of stone (8, 26). Salt bridges primarily influence the formation and growth of struvite crystals. Salt bridges are formed by combining two types of noncovalent interactions: hydrogen bonding and electrostatic interactions. When ions like NH4+ and PO4^3- are present in water, they enhance hydrogen bonding, potentially forming salt bridges. The effect of salt bridges is closely linked to the mechanisms of biomineralization, which explain the process of struvite crystal formation. Hydrogen bonds play a crucial role in regulating the crystallization of complex salts, affecting both the initiation and growth of crystals and the resulting arrangement of ions within the crystal lattice that influences the morphology of the crystals (27, 28).

## Potential role of microorganisms in infection stones

The formation of infection stones presents a significant challenge due to their potential for rapid growth. These stones primarily consist of struvite, a combination of bacteria, crystals, and a protein matrix, often accompanied by CaP and CaOx (9). In the United States, struvite accounts for 5% to 15% of renal stones; globally, it contributes to 30% of nephrolithiasis cases. Developed countries typically have a lower prevalence of struvite stones, representing only 4% of urinary stones, while in developing countries, it’s about 10–20% of urinary stones (29). The exact prevalence of struvite stones in the Middle East is unknown, but it is believed to be higher due to several region-specific risk factors compared to the rest of the world. These factors include obesity, hot and dry weather, and dietary habits that may contribute to the formation of infection-related stones. Patients with infection stone represented a high prevalence of recurrent stones (61.1%) (30, 31).

Struvite stones develops in the kidneys or other parts of the urinary tract due to infection with urease-positive microorganisms. The formation of these stones appears to be heavily influenced by the composition of urine and the interaction between bacteria and various components of the urinary system (29). Bacteria in infected urine produce an enzyme called urease, which breaks down urea into ammonia and carbon dioxide that results in alkaline environment, forming struvite stones. Another way in which bacterial infection can contribute to stone formation involves an increase in crystal adherence (32). A study by Parsons et al. demonstrated that ammonium can disrupt the glycosaminoglycan layer that typically covers the bladder mucosa, facilitating bacterial attachment to the mucosal surface (33). It is plausible that a similar process occurs in the renal collecting system when affected by bacterial infection, potentially causing damage to the glycosaminoglycan layer. This, in turn, can enhance bacterial adhesion, trigger tissue inflammation, stimulate the production of organic matrix, and promote interactions between crystals and the matrix (34).

However, not all patients with urease-positive infections develop struvite stones, emphasizing the importance of understanding urine chemistry and other factors unrelated to bacteria in forming these stones (35). Additionally, it is essential to evaluate the risk factors associated with struvite stone formation, as they influence the likelihood of recurrence or growth and guide pharmacological treatments (36). Identified risk factors for the development of struvite stones include being female, having congenital urinary tract abnormalities, upper age, experiencing urinary obstruction or diversion, diabetes, using indwelling catheters, neurogenic bladder, medullary sponge kidney, or distal renal and tubular acidosis.

In a study conducted by Zhang et al., 115 out of 1520 stones (7.6%) were classified as infection stones, and it was noted that this type of stone occurred more frequently in females (37). Another study involving 1204 patients with renal stones from 12 institutions across 10 different countries reported that among these patients, 56 (4.6%) had struvite stones, while 15 (1.2%) had carbonate apatite (carbapatite) stones. The incidence of struvite stones varied among the different countries. The lowest occurrence was observed in Canada, Iraq, Argentina, China, Poland, and Italy, at 22%, 3%, 3%, 3%, 3%, and 3.5%, respectively. Intermediate rates were reported in Bulgaria (5.4%) and Egypt (5.5%). In contrast, Pakistan (18%) and India (23%) had a more significant rate of struvite stones (38).

According to a study conducted by Kumar et al., it was found that about 74.77% of stones in India were a combination of CaOx and CaP, resulting in a mixed composition of these two components. Uric acid stones accounted for 12.2% of the cases, followed by struvite stones in 11.22% cases and cysteine stones in 1.87% cases. An interesting observation was made that out of patients with preoperative positive urine culture, 53.33% had both calcium oxalate and calcium phosphate stones, 40% had struvite stones, and 6.67% had uric acid stones (39). Stones with a struvite content exceeding 80% were consistently found to harbor urea-splitting bacteria, while stones with 20% struvite content predominantly contained non-conventional urease-producing bacteria (30). Recent studies investigating bacterial morphology in infection stones have indicated an increasing prevalence of non-urease-producing bacteria. A study by Paonessa et al. found that 23% of urine samples from patients treated with percutaneous nephrolithotomy and having struvite stones contained non-urease-producing bacteria (40, 41).

Analyzing struvite stones that had a composition of over 50% struvite, Parkhomenko et al. discovered that only half of the positive stone cultures were derived from bacteria capable of producing urease. The isolation rates for *Escherichia coli* (*E. coli*) and *Enterococcus* were 18% and 12%, respectively. The higher occurrence of infections in patients with infection stones, particularly involving *E. coli*, can be attributed to its frequent presence due to its short replication time. Additionally, studies have suggested the potential transfer of the urease gene through plasmids (42, 43). Among females, there is a higher prevalence of CaP and struvite stones, with struvite stones common in younger and older age groups. Stones of metabolic origin, such as cystine stones, are more frequent at younger ages (44).

Kidney stones were more commonly observed in males within the adult population. In the past decade, there has been a consistent prevalence of stones in men, with rates of 11.6% during 2007–2008 and 11.9% during 2017–2018. However, there has been an increase in stone prevalence among women, rising from 6.5% to 9.4% during 2017–2018 (45). The study conducted by Ranji et al. reported a male-to-female ratio of 1.3:1, which aligns with previous findings suggesting a higher incidence of stone formation in males (46). Seitz et al.’s study also indicated a rising trend in kidney stone prevalence, with men being more susceptible. The higher occurrence of stones in men may be associated with elevated levels of androgens, which could contribute to the formation of calcium oxalate stones in the urine (47). Meanwhile, according to the study by Xierzhati Aizezi et al., infection stones were more prevalent in females (12). On the other hand, the increased prevalence of stone infections in females may be linked to anatomical factors of the female urethra. The shorter length of the female urethra, located close to the vagina, makes it more susceptible to colonization by pathogenic bacteria such as *E. coli*. This increases the likelihood of UTIs and subsequent formation of infectious stones in women (48, 49).

## Potential role of microorganisms in non-infection stones

Metabolic disorders are commonly attributed to calcium-based stone formation, but the potential role of microorganisms in this process has not been thoroughly explored. Evidence suggests a possible association between bacteria and urinary stones, particularly those composed of CaOx, CaP, or a combination of these with other stone types (26). Stone cultures from specific urinary stones have tested positive, indicating the presence of bacteria. Previous studies have reported positive cultures in 13% to 44% of CaOx stones. The most frequently identified bacteria in these stone cultures were *E. coli* (15–35%), *Pseudomonas* spp., and *Proteus*, known to be urease-producing bacteria often associated with the development of struvite stones (10). For example, in a study involving 100 patients with urolithiasis, positive stone cultures were observed in 16%, 15%, 85%, 61%, and 20% of pure CaOx stones, CaOx-CaP stones, pure struvite stones, struvite-CaOx stones, and pure CaP stones, respectively. These findings highlight the potential involvement of bacteria in forming different types of urinary stones and underscore the need for further research in this area (26).

The bacteria most commonly detected in calcium-based stones include *E. coli*, *Pseudomonas* spp., and other proteolytic microorganisms typically found in struvite stones. Notably, an *in vitro* study revealed that *E. coli* cells could reduce urinary citrate levels, strongly associated with increased calcium precipitation (50). This suggests that urease-induced CaP crystallization may be enhanced by this phenomenon. Bazin et al. conducted research and observed a significant presence of bacterial imprints in carbapatite stones, while no such imprints were found in struvite stones. In mixed stones, such as those containing both struvite and carbapatite, bacterial imprints were primarily observed in tiny carbapatite crystals rather than large struvite crystals (26, 51).

Carpentier et al. proposed that bacterial imprints indicate the occurrence of previous or ongoing UTIs involving both urea-hydrolyzing and non-urea-hydrolyzing bacteria, which are associated with the formation of calcium phosphate stones (52). This study observed a positive association between bacterial imprints, amorphous carbonated CaP (ACCP), and a high carbonation rate (carbonate: phosphate ion ratio) in carbapatite stones without struvite. Furthermore, it has been reported that both ureolytic and non-ureolytic bacteria found in human urine can form calcium crystals within their cells, which could serve as additional sites for stone formation (26, 52). Chutipongtanate et al. studied the lithogenic potential of Gram-negative and Gram-positive bacteria on CaOx. The researchers utilized morphological evaluation, a new screening method, and gold-standard assays to demonstrate that bacteria can directly promote the growth and aggregation of CaOx crystals. Specifically, CaOx crystals were observed in the presence of two uropathogenic bacteria, *E. coli* and *K. pneumoniae*. Additionally, non-uropathogenic bacteria such as *S. aureus* and *S. pneumoniae* exhibited lithogenic effects on CaOx (53).

In a case study conducted by Wu et al., a 62-year-old male patient presented with symptoms including haematuria, fever, and flank pain. Upon thorough examination, the patient was diagnosed with acute pyelonephritis. Computed tomography scans revealed the presence of a partial staghorn stone in the left kidney. Notably, both the culture of the surgical lesion and the urine culture obtained during the initial visit yielded *Citrobacter koseri*. Furthermore, stone analyses performed as part of the diagnostic process indicated that the stone was primarily composed of CaOx (28). Halinski et al. investigated carbapatite stones and ammonium urate stones, finding that *E. coli*, Gram-positive bacteria, and *Klebsiella* spp at 47.6%, 14%, and 7.8%, respectively. *Proteus* spp. and *P. aeruginosa* accounted for only 4.6% and 2.3% of cases, respectively. Within the Gram-positive group, the identified uropathogens included *Enterococcus faecalis* (*E*. *faecalis*), *E. faecium*, *S. aureus*, *S. cepra*, *S. agalactiae*, and other rare Gram-positive bacteria. Additional isolates included other Gram-negative bacteria, *Candida* spp., *Ureaplasma urealyticum*, and mixed flora (38).

In another study by Wang et al., the incidence of UTIs in the group with infection stones was significantly higher compared to the groups with CaOx and uric acid stones. Positive urinary cultures were found in 29.1% of cases among the infection stone group, significantly higher than the rates observed in the CaOx group (17.46%) and the uric acid stone group (23.83%). The most commonly identified bacteria were *E. coli*, *Proteus mirabilis*, *S. agalactiae*, as well as *Klebsiella*, *Pseudomonas*, *E. faecalis*, *E. faecium*, *S. epidermidis*, *P. aeruginosa*, *S. aureus*, *S. haemolyticus*, and *Enterobacter cloacae* (30). Lemberger et al. discovered that the highest levels of confidently detectable bacterial colonization were observed in Apatite and Apatite/CaOx/CaPhos stones (54).

## Urinary microbiome and urinary stones

While multiple factors influence nephrolithiasis, lifestyle significantly impacts its development. Another important factor to consider is the role of an imbalanced microbiome (55, 56). In a scientific context, the term “microbiome” refers to the collection of microorganisms that exist in symbiotic, commensal, or pathogenic relationships within various regions of the body, such as the skin, oral cavity, respiratory tract, gastrointestinal tract, urinary tract, reproductive tract, and more (57, 58). These microorganisms can have positive, negative, or neutral effects on human health, and the interactions within the microbiome are crucial. The gut microbiota actively influences the metabolism of substances and energy. Extensive research has shown the existence of a cross-talk between the gut microbiome and the kidneys. For instance, patients with chronic kidney disease often exhibit disruptions in their intestinal ecology (59–61).

The relationship between the urinary microbiome and urinary stone disease (USD) is a burgeoning area of research that offers insights into the pathogenesis and potential management of this common condition. Traditionally, the urinary tract was considered sterile, but recent advancements in molecular techniques have revealed the presence of a diverse microbial community within the urinary system, known as the urinary microbiome (10). Certain microbial species within the urinary tract may influence stone formation by modulating the chemical environment of the urine.

Moreover, the urinary microbiome may interact with the host immune system, affecting inflammatory responses within the urinary tract. Chronic low-grade inflammation has been associated with stone formation and recurrence. Dysbiosis, or an imbalance in the urinary microbiome, could potentially exacerbate inflammation and contribute to stone formation (62).

Conversely, some microbial species within the urinary microbiome may confer protective effects against urinary stone formation. These beneficial bacteria may compete with stone-forming pathogens for resources or produce metabolites that inhibit crystal formation. Understanding the complex interplay between the urinary microbiome and urinary stone disease could lead to novel therapeutic approaches, such as probiotics or targeted antimicrobial treatments, aimed at restoring microbial balance and preventing stone formation (54, 63).

The potential involvement of the gut microbiome in the pathogenesis of KSD has become a subject of increasing scrutiny in recent research. Significantly different alterations in the gut microbiota have been observed between individuals afflicted with renal calculi and those without such conditions. Notably, studies have revealed a notably higher abundance of Bifidobacterium in individuals classified as “normal” in comparison to those with KSD. Furthermore, it is posited that gut bacteria may confer protective effects against the formation of the predominant type of kidney stone, CaOx stones. CaOx stones are renowned as the most prevalent form of kidney stones, with oxalate detected in approximately 75% of urinary calculi cases (64, 65). Oxalate, a byproduct of amino acid metabolism, necessitates renal elimination due to its toxic nature. Epidemiological investigations and animal models have provided evidence of a correlation between heightened oxalate excretion and the propensity for kidney stone development, thereby suggesting that dietary reduction of oxalate and calcium may mitigate associated risks (5).

The human body cannot produce enzymes that can break down oxalate, primarily due to excessive dietary exposure and its toxic nature. Instead, a variety of bacteria present in our gut contribute to the degradation of oxalate. Several studies have specifically investigated the oxalic acid-degrading capabilities of bacteria such as *Oxalobacter formigenes* (*O. formigenes*), *Bifidobacterium*, *Lactobacillus*, and *O. formigenes*, in particular, can utilize oxalate as a carbon and energy source (66, 67). This bacterium has been identified as potentially playing a role in reducing the recurrence of CaOx kidney stone formation. Every day, approximately 51% of dietary oxalate is degraded by this bacterium, which also helps regulate oxalate transport in the intestines (68). Consequently, *O. formigenes* reduces the amount of oxalate absorbed by the colon, decreasing its excretion by the kidneys. Investigations demonstrated that oxalate stones result in higher urine oxalate excretion and lower levels of *O. formigenes*. This suggests that the lack of colonization by *O. formigenes* may be associated with the formation of oxalate stones (69).

**FIGURE 2:**
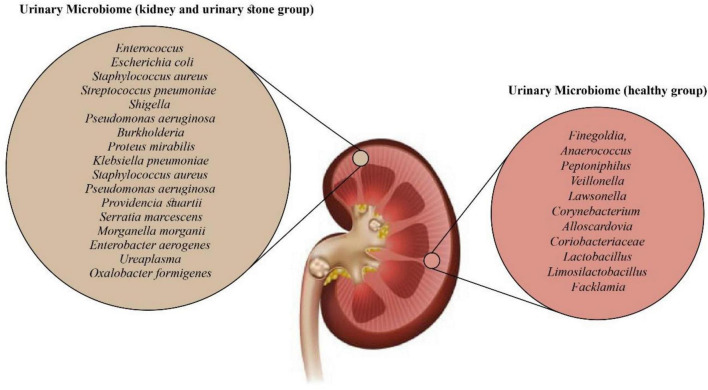
Urinary microbiome related with urolithiasis (10, 54, 73–75).

Investigating the association between gut microbiota and urinary stone formation may offer new targets for preventing and treating upper urinary urolithiasis. Additionally, this particular species of bacteria is sensitive to commonly used antibiotics and can reduce urinary oxalate levels when administered orally (70). Studies suggest that dysbiosis, resulting from a loss of function within this bacterial species, may contribute to the formation of CaOx stones (71). The study conducted by Zampini et al. sheds light on the nature and location of dysbiosis associated with USD. According to their findings, Lactobacillus and Enterobacteriaceae exhibit both protective and pathogenic roles in USD, which may not always be detectable through conventional culture-based methods of bacterial analysis in urine and kidney stones. Compared to the gut microbiome, antibiotics can lead to long-term alterations in the microbiome, increasing the risk of developing USD (Figure 2) (72).

Using Mendelian randomization, Zhang et al. applied the inverse variance-weighted (IVW) method. The results obtained from the IVW analysis confirmed that specific microbial classifications, such as class Deltaproteobacteria, order NB1n, family Clostridiaceae *1*, genus *Barnesiella*, genus *Clostridium sensu_stricto_1*, genus *Flavonifractor*, genus *Hungatella*, and genus *Oscillospira*, displayed a protective effect against upper urinary urolithiasis. Conversely, *Eubacterium xylanophilum* exhibited an unfavorable effect, but no outlier single nucleotide polymorphisms (SNPs) were found. The study established an association between several genera and upper urinary urolithiasis. Nevertheless, further validation of these findings is still required through randomized controlled trials (70).

The urobiome, the human urinary microbiome, is vital in maintaining urogenital homeostasis. Microbiome imbalance can contribute to various urological conditions, including UTIs, voiding disorders, tumorigenesis, nephrolithiasis, and recurrent stone disease. However, no specific bacterial species has been identified as the sole cause of stone formation thus far. Through advanced detection technologies, studies have identified *Corynebacterium*, *Lactobacillus*, and *Ureaplasma* bacteria as members of the urinary microbiota (54). The urinary microbiome exhibits lower diversity and a smaller population than other microbiomes, such as the gut and skin (76, 77). Despite its proximity to the bladder, the urinary microbiota differs from populations found in the gut and vagina (73). Our understanding of their role as urinary microbiota in maintaining health and predisposing to diseases and disorders is still in its early stages. Nonetheless, the urinary tract provides a niche environment for microbes, and expanding our knowledge of the urinary microbiome holds promise for better understanding urinary system diseases (76).

For example, hosts provide nutrition, regulate PH levels, and supply oxygen to the bacteria that make up the urinary microbiome. In return, these microbes assist in establishing a resilient immune system in the urinary tract. Disruption of resident microbiomes has been demonstrated to contribute to host health issues, including neoplasms, UTIs, bacterial vaginosis, and inflammatory bowel disease (77). In the context of urolithiasis development, a decrease in bacterial diversity can indicate dysbiosis, potentially leading to stone formation (78). A single-center observational study by Lemberger et al. revealed that patients with metabolic syndrome had a distinct stone microbiome with a notable increase in *E. coli*, *Shigella, Klebsiella*, Enterococcaceae*, Proteus*, and *Sphingomonas*. On the other hand, individuals without metabolic syndrome had stones affected by *Ureaplasma* and Staphylococcaceae (54). Bacterial presence in the stones resulted in a more extended hospital stay and severe outcomes. At the same time, no direct association was reported between the types of bacterial genus and kidney stone formation. However, it demonstrated that pathogenic Enterobacteriaceae were highly prevalent in all types of stones, indicating an association between these bacteria and struvite stones, CaOx, and calcium phosphate stones (54).

Stern et al. reported that in the group with kidney stones, *Bacillus* was more prevalent (3.4 times), while *Prevotella* was 2.8 times more abundant in the group without stones. One interesting finding was that certain microbial producers of short-chain fatty acids showed a decreased proportion among patients with nephrolithiasis, as observed in an observational study by Liu et al. (79, 80). Another study by Shen et al. focused on analyzing the midstream urine of patients with CaOx stones. They discovered two distinct microbiome clusters in this population exhibiting significant beta diversity. The first cluster primarily consisted of *Acinetobacter*, *Pseudomonas*, and *Enterococcus* bacteria and displayed lower urinary white blood cells per high-power field (WBC/HP) index (81). In contrast, the second cluster had a higher WBC/HP index and a greater frequency of *E. coli, Klebsiella pneumoniae* (*K. pneumoniae*), and *Salmonella enterica*, indicating an increased potential for severe infections (81). The finding from an investigation to evaluate the urine microbiome of patients experiencing acute urinary retention due to stones or tumors showed an abundance of *Pseudomona*s, *Acinetobacter*, and *Sphingomonas* bacteria, as well as an underrepresentation of *Lactobacillus, Streptococcus, Gardnerella, Prevotella*, and *Atopobium* (82). Additionally, *Pseudomonas aeruginosa* (*P. aeruginosa*) has been associated with an increase in urine PH and the rate of crystallization of CaOx, which are metabolic pathways favoring stone formation (83, 84). Despite advancements in technology that provide a better understanding of the role of bacteria in lithiasis, the question of whether UTIs promote stone formation or vice versa remains unanswered (74).

A case-control study comparing the blood, urine, and stool microbiomes of patients with USD and healthy controls discovered that the gut bacteria including *Collinsella, Peptostreptococcus, Sutterella, Barnesiella, Peptococcus, Senegalimassilia, Butyricimonas, Bilophila, Ruminiclostridium-9, Coprobacter, Mogibacterium*, and *Cupriavidus* were significantly different among genders (85). Likewise, in a study by Miller et al., gender-specific differences were observed in the beta diversity (diversity between different samples) but not the alpha diversity (diversity within individual samples) of the gut microbiome in patients with and without USD (86). The gender-specific microbiome variations could account for the differences in USD. In another study by Ellison et al., to compare the microbiome signals in children with initial and recurrent nephrolithiasis and explore additional associations in microbiome composition and diversity within this population, the potential indications of lower microbial diversity and oxalate gene expression in pediatric kidney stone patients with recurrent episodes were observed (75). These findings suggest further investigation to determine their potential as diagnostic markers for future kidney stone events (75). In another study by Liu et al., the urinary microbiota composition of urolithiasis patients was compared to that of healthy individuals to identify potential microbial markers and their association with clinical parameters. The study revealed that the urinary microbiota composition of urolithiasis patients differed significantly from that of healthy controls. Certain microbial taxa, such as Ruminococcaceae and Proteobacteria, showed promise as potential biomarkers for urolithiasis. These findings open avenues for further research into the role of microbiota in urolithiasis and the development of microbiome-based therapeutic strategies (71).

## UTI in patients with urolithiasis

UTI is a commonly observed condition in cases of urolithiasis, and recent studies have indicated that 7–28% of individuals with KSD also have concurrent UTIs. The risk of females developing KSD in association with UTI is four times higher than that of males, and this condition is linked to an increased risk of sepsis (87). The association between KSD and urinary tract infections has yet to be fully understood, as it remains unclear whether one condition is the cause or consequence of the other. When patients present with KSD accompanied by urosepsis, immediate surgical decompression is necessary, followed by planned stone treatment once the infection has been resolved. UTI has been implicated in the formation of kidney stones (87). Persistent infections caused by bacteria that produce urease can lead to the development of infection stones composed of monoammonium urate, struvite, and/or carbonate apatite, which complicates the treatment of urolithiasis. Some complications, including asymptomatic bacteriuria, UTI, and sepsis, have been observed following treatment with extracorporeal shock-wave lithotripsy (88).

Patients who undergo percutaneous nephrolithotomy (PCNL) for severe or multiple stones may experience postoperative systemic inflammatory response syndrome. A small percentage of these patients may progress to urosepsis, which can have serious consequences such as septic shock (88). Among all urogenital tract infections, pyelonephritis is the most severe and can lead to dangerous complications. In cases of persistent urinary tract infection caused by bacteria that produce urease, the PH of the urine increases, creating favorable conditions for the formation of infection stones (89). In infection stones where more than 80% of the composition is struvite/apatite, the UTIs are predominantly associated with urease-producing pathogens. Proteus, Morganella, and Providencia spp. are the most frequent bacteria in this category, whereas *Klebsiella, Pseudomonas*, and *S. aureus* spp. may have varying levels of urease production and are less frequently associated with stone formation (90). Furthermore, it has been established that urinary tract obstruction is a risk factor for UTIs and the development of infection stones. When urine flow is impeded due to obstruction, the risk of infection increases as the urine cannot pass smoothly. Additionally, individuals with multiple stones are more susceptible to infections than those with a single stone. This is likely because multiple stones have a higher likelihood of causing obstruction, which can lead to urinary retention and significantly increase the chances of UTIs (88).

A study by Zhang et al. found that patients with upper urinary tract stones had significantly higher rates of CaOx stones, while those with lower urinary tract stones were more likely to have infection stones. Patients with UTIs had a higher prevalence of infection stones, whereas patients without UTIs had a higher incidence of CaOx stones. The study also revealed a correlation between the rate of infection stones and UTI and a higher urine PH profile (37). In another study by Yongzhi et al., the prevalence and etiology of UTIs in patients with urolithiasis were investigated, and results showed that about 22.0% had UTIs. Gram-negative bacilli were the most commonly isolated pathogens, accounting for 93.3% of cases, followed by gram-positive bacilli, which accounted for 4.5%. Among the gram-negative bacilli, the most prevalent was *E. coli*, accounting for 52.8% of cases, followed by *P. aeruginosa* at 15.16%, *K. pneumoniae* at 12.35%, and *P. mirabilis* at 3.93%. Patients with multiple stones had a higher infection rate than those with a single stone (41.3% vs. 16.0%) (88).

Furthermore, Kumar et al. reported that 67% of the patients exhibited symptoms of UTI, with the most common symptom being severe groin pain. The bacteriological profile of the patients with UTIs showed that *E. coli* was the predominant pathogen, accounting for 54.3% of cases, followed by *K. pneumoniae* at 19.6%, *Enterococcus* species at 8.7%, *P. mirabilis* at 6.5%, *S. aureus* at 6.5%, *C. koseri* at 2.2%, and *P. aeruginosa* at 2.2% (39). Another study reported that among the patients with struvite stones, 64.3% had positive urine cultures, while among patients with other types of stones, 26.7% had positive urine cultures. There was a notable difference in the bacterial pathogens observed between patients with struvite stones and those with other stones. Among the detected isolates in patients with struvite stones, the most commonly identified pathogens were *E. coli*, *Proteus* species, *Klebsiella* species, Gram-positive bacteria, and *P. aeruginosa* as 7.7%, 27.7%, 16.7%, 5.5%, and 5.5%, respectively (38). A recent meta-analysis confirmed that a stone culture and renal pelvic urine culture are more reliable than a midstream urine culture for identifying the microorganisms and selecting antibiotic therapy for UTIs after PCNL (91).

Furthermore, a negative culture result can be attributed to urease-producing bacteria (such as *Ureaplasma urealyticum* and *Corynebacterium urealyticum*) that may not grow in standard urine cultures (92). In a study by Rizwan et al., about 24.33% of patients were diagnosed with UTIs. The most common pathogens were gram-negative, gram-positive, and fungi, accounting for 90.41%, 5.47%, and 4.10% of cases, respectively. Among the gram-negative bacilli, *E. coli* was the predominant pathogen, making up 53.42% of cases, followed by *P. aeruginosa*, *K. pneumoniae, P. mirabilis*, and other bacteria, which accounted for 15.06%, 12.32%, 4.10%, and 5.47% of cases, respectively. The study concluded that gender, age, urinary tract blockages, stone structure, and the presence of multiple stone locations could be considered independent risk factors for UTIs in patients with urolithiasis. However, no statistically significant relationship was found between drinking and smoking habits and the incidence of UTIs (93).

## Association of kidney stones and recurrent UTIs

UTIs play a significant role in the formation of infection stones, specifically struvite stones, often accompanied by CaOx or calcium carbonate apatite, due to the urea-splitting mechanism of urease-producing gram-positive and gram-negative bacterial species such as *Proteus*, *Staphylococcus, Pseudomonas, Providencia, Ureaplasma*, and *Klebsiella* (87). Kidney stone formation is associated with various metabolic disorders that result in altered urinary excretion levels of calcium, uric acid, and oxalate and reduced citrate excretion. These metabolic syndromes contribute to the development of kidney stones (4). Furthermore, bacteria also play a role in developing CaOx, CaP, and struvite stones. UTIs are primarily caused by various bacteria, mainly from the Enterobacteriaceae family. UTIs have been implicated in the development of kidney stones. Patients who have KSD along with recurrent UTIs or positive symptomatic urine culture often require complete removal of their stones to effectively treat their UTIs. The global incidence of KSD has been on the rise, with a lifetime prevalence reaching up to 14% (4, 87).

Epidemiological data confirms a growing trend of the lifetime prevalence of KSD to about 14%. Additionally, at least 50% of patients will experience a recurrence of stones within ten years (94). Any urinary calculi associated with an infectious agent can lead to recurrence. Ripa et al. conducted a systematic review to examine the association between KSD and UTIs. The findings of the study provide support for the existing evidence that patients with KSD often experience recurrent or concurrent UTIs (36). Interestingly, inconsistent results were observed when comparing the risk of UTIs based on different stone compositions. Some studies demonstrated unexpectedly higher risks of UTIs in cases of CaOx and uric acid stones or when CaOx stones were mixed with phosphate, magnesium ammonium phosphate, and uric acid stones (95). Findings suggest that colonized urine or stone samples were not associated with struvite and infection stones but may involve almost all chemical stone compositions. In a study conducted by Heidari et al., it was observed that patients with renal stones had a higher incidence of recurrent UTIs than the control group (96). Preoperative urine culture yielded positive results in 79% of the patients, and 21% had a history of recurrent UTIs about 79% of them represented the associated risk factors, including intermittent catheterization, DM, and contralateral stones. It has been reported that in pediatric patients under the age of 2 diagnosed with nephrolithiasis, the presence of metabolic risk factors and stone size is significantly associated with the occurrence of both single and recurrent UTIs (97, 98).

## Bacterial biofilm formation in UTI

Forming biofilms is a fundamental aspect of UTIs, particularly in catheter-associated UTIs (CAUTIs), which account for 40% of all hospital-acquired infections. A biofilm is a complex structure formed by bacteria encased in a self-produced matrix of substances such as exopolysaccharides. Bacterial biofilms play a crucial role in these infections and significantly contribute to the high recurrence rates and antimicrobial resistance in UTIs (11, 99, 100). In addition to raising urinary PH to promote the crystallization of magnesium ammonium phosphate, ammonia production also contributes to the development of struvite stones. This occurs by damaging the protective glycosaminoglycan layer that covers urothelial cells, leaving them vulnerable to bacterial pathogens (89, 101). Biofilms can be found in various urological settings, including catheters (urethral, continuous ambulatory peritoneal dialysis, and hemodialysis catheters) and kidney stones. These biofilms contribute to various complications, including pyelonephritis, cystitis, the formation of staghorn stones, and catheter encrustation (102).

In the context of UTIs and struvite stone formation, after breaching the protective barrier, bacteria can attach to the surface of the urothelium (the lining of the urinary tract) and establish a bacterial biofilm (103). Previous studies have shown that struvite stones have a three-dimensional structure composed of many bacteria embedded within the biofilm matrix. The matrix primarily comprises exopolysaccharides secreted by the bacteria during biofilm formation (104, 105). Studies found that urease activity, an enzyme the bacteria produces, plays a significant role in struvite stone formation within the bacterial biofilm. Urease breaks down urea present in the urine, resulting in the release of ammonia. This localized increase in ammonia concentrations within the biofilm leads to a rise in PH levels. The higher PH facilitates the crystallization of magnesium ammonium phosphate, a significant component of struvite stones (103).

The extracellular polymeric substance (EPS) matrix within the biofilm can serve as a site for crystal nucleation. The biofilm formation process and subsequent struvite biomineralization follow some steps (106, 107). First, ureolytic microorganisms attach to the urinary tract, forming a thin layer of planktonic cells and urinary metabolites. This attachment is followed by the formation of microcolonies, which is the initial phase of biofilm development accompanied by the production of EPS. Ureolysis by these microorganisms increases the PH and the concentrations of NH4+ and CO3^2- in the urine. Alkaline conditions and the elevated levels of NH4+ and CO3^2- in the urine lead to primary crystals, including carbapatite, struvite, and possibly calcium carbonate (108). These crystals can become trapped within the EPS matrix, and detachment of microbes from the biofilm may occur. Crystals grow and aggregate around the attached bacteria within and outside the EPS matrix. Planktonic bacteria adhere to pre-existing crystals, forming more microcolonies that precipitate minerals. The process results in forming layers consisting of bacteria encased in minerals. The aggregation of crystals is influenced by organic macromolecules present in urine, which can either inhibit or promote crystal aggregation (107).

Since the common microorganisms found in kidney stone biofilms, such as *P. aeruginosa*, *E. coli*, *K. pneumoniae*, *Proteus mirabilis*, various *Staphylococcus* species, and *Enterococcus* species, some possess urease activity and contribute to mineral formation in kidney stones. *Proteus mirabilis* is notably associated with CAUTIs in patients with long-term urinary catheters. It forms crystalline biofilms that enable its colonies to survive in challenging environments. This is concerning due to the high antimicrobial resistance (AMR) exhibited by biofilm-associated bacteria, which is 10–1,000 times higher than their non-biofilm counterparts. The ability of *Proteus* to form biofilms is attributed to several important virulence factors, including swarming motility, fimbriae (surface appendages), urease production, capsule polysaccharide, and efflux pumps (109).

Furthermore, *P. mirabilis* plays a distinct role in individuals who undergo chronic catheterization, as it is closely linked to the formation of urinary stones, which is a challenging complication to manage. This is particularly significant for patients with spinal cord injury (between 20 and 50%) who rely on long-term indwelling urinary catheters for bladder management (103). Consequently, these patients face increased susceptibility to CAUTIs and other complications, including stone formation and catheter obstruction caused by the accumulation of proteinaceous and mineral deposits. The presence of *P. mirabilis* exacerbates the risk and complexity of these complications, highlighting the need for effective management strategies in individuals with chronic catheterization.

## Conclusion

**FIGURE 3:**
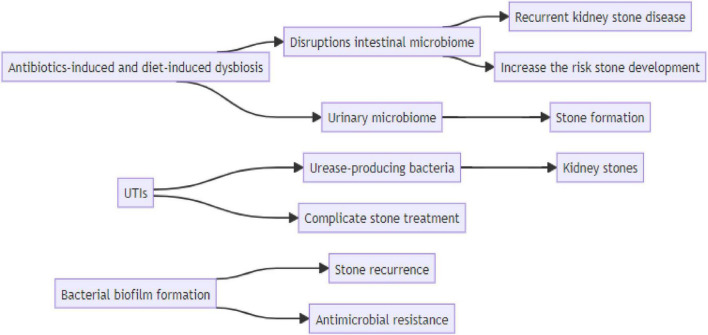
Schematic diagrams illustrate the connection between risk factor and kidney stone disease.

The association between bacteria and urinary stones in patients with urolithiasis is a complex and evolving area of research. The urinary microbiome has emerged as a significant factor in stone formation, and disruptions in the gut microbiota can increase the risk of stone development. Additionally, emerging evidence suggests a potential role of microorganisms in the formation of non-infection stones, particularly those composed of calcium oxalate. The relationship between UTIs and kidney stones is complex, and UTIs can complicate stone treatment, especially when urease-producing bacteria are involved. Bacterial biofilm formation contributes to stone recurrence and antimicrobial resistance, particularly in catheter-associated UTIs. Unraveling the association of bacteria and urinary stones in patients with urolithiasis is a dynamic field with important clinical implications, Figure 3. Continued research efforts will enhance our understanding of the pathophysiology of stone formation, aid in developing targeted therapies, and ultimately improve the management and outcomes for individuals affected by urolithiasis.
